# The Resistance Phenotype and Molecular Epidemiology of *Klebsiella pneumoniae* in Bloodstream Infections in Shanghai, China, 2012–2015

**DOI:** 10.3389/fmicb.2017.00250

**Published:** 2017-02-23

**Authors:** Shu-zhen Xiao, Su Wang, Wen-man Wu, Sheng-yuan Zhao, Fei-fei Gu, Yu-xing Ni, Xiao-kui Guo, Jie-ming Qu, Li-zhong Han

**Affiliations:** ^1^Department of Clinical Microbiology, Ruijin Hospital, Shanghai Jiao Tong University School of MedicineShanghai, China; ^2^Department of Clinical Laboratory, Ruijin Hospital, Shanghai Jiao Tong University School of MedicineShanghai, China; ^3^Department of Clinical Laboratory, Xiangya Hospital, Central South UniversityChangsha, China; ^4^Department of Medical Microbiology and Parasitology, Institutes of Medical Sciences, Shanghai Jiao Tong University School of MedicineShanghai, China; ^5^Department of Pulmonary Medicine, Ruijin Hospital, Shanghai Jiao Tong University School of MedicineShanghai, China

**Keywords:** *Klebsiella pneumoniae*, bloodstream infections, resistance phenotype, molecular epidemiology, extended-spectrum β-lactamase

## Abstract

*Klebsiella pneumoniae* (*K*.pneumoniae) is a common nosocomial pathogen causing bloodstream infections. Antibiotic susceptibility surveillance and molecular characterization will facilitate prevention and management of *K. pneumoniae* bloodstream infections. *K. pneumoniae* isolates causing bloodstream infections were consecutively collected between January 2012 and December 2015 in Shanghai. Eighty isolates (20 per year) were randomly selected and enrolled in this study. Drug susceptibility were determined by the disk diffusion method. Polymerase chain reaction (PCR) was employed to detect extended-spectrum β-lactamases (ESBLs), carbapenemases, and seven housekeeping genes of *K. pneumoniae*. eBURST was used for multi-locus sequence typing (MLST). More than 50% isolates were resistant to cefuroxime, ampicillin-sulbactam, and piperacillin, while carbapenems had lower resistant rates than other antibiotics. Of the 80 isolates, 22 produced ESBLs, and 14 were carbapenemase producers. In the ESBL-producing *K. pneumoniae* isolates, the most common ESBL genes were *bla*_SHV_ and *bla*_CTX−M_. Thirteen carbapenemase producers harbored *bla*_KPC−2_ and one other carried *bla*_NDM−5_. ST11 (14/80) was the most frequent sequence type (ST), followed by ST15 (7/80) and ST29 (4/80). Our data revealed high prevalence of antibiotic resistant *K. pneumoniae* isolates from bloodstream infections but their genetic diversity suggested no clonal dissemination in the region. Also, one *K. pneumoniae* isolate harbored *bla*_NDM−5_ in this study, which was firstly reported in Shanghai.

## Introduction

*Klebsiella pneumoniae* (*K. pneumoniae*) can cause ventilator-associated pneumonia, urinary tract infection, sepsis, catheter-related infection, and bacterial meningitis (Tumbarello et al., 2006; Du et al., 2014), and is also the most common causative gram-negative bacterium in nosocomial and community-acquired infections (Hu et al., 2015). Unfortunately, extended and overuse of antibiotics may potentiate antibiotic resistance of *K. pneumoniae* strains to cephalosporins, aminoglycosides, fluoroquinolones, and even carbapenems (Guana et al., 2014), which will be difficult and costly to control.

Extensive use of invasive procedures and glucocorticoids has increased the incidence of bloodstream infections and caused high mortality in patients (27.2–40.8%; Marra et al., 2006; Yang et al., 2010; Lv et al., 2014; Wang et al., 2016). Of the gram negative bacilli implicated in nosocomial bloodstream infections, *K. pneumoniae* was one of the most common pathogens, second only to *Escherichia coli* (Ghadiri et al., 2012; Lv et al., 2014). The prevalence of *Klebsiella* spp. bloodstream infections was 7.6% in the United States, Canada, South America, and Europe according to SENTRY (Biedenbach et al., 2004). On the basis of a large-scale survey from China, *K. pneumoniae* caused 11.3% of all bloodstream infections in 2011–2012 (Lv et al., 2014).

The wide dissemination of drug resistant pathogens leads to their increasing prevalence in bloodstream infections. Data from China showed that 53.3% *K. pneumoniae* isolates were multidrug-resistant (Lv et al., 2014). In Europe, Latin America, and North America, 21.7, 42.7, and 5.8% of *Klebsiella spp*. had the extended-spectrum β-lactamase (ESBL) phenotype (Biedenbach et al., 2004), while as high as 60.6% *K. pneumoniae* isolates in Greece were carbapenemase producers (Daikos et al., 2014). And the most prevalent group of carbapenemases was *K. pneumonia* carbapenemase (KPC). Studies have shown that the drug-resistant organisms bloodstream infection was one important risk factor for mortality and negatively impacted the treatment outcome of patients (Kim et al., 2002; Tumbarello et al., 2006). Limited data on susceptibility and molecular epidemiology of *K. pneumoniae* causing bloodstream infections were available in Shanghai. In the study, we have monitored resistance phenotype, the prevalent resistant genes and sequence types (STs) of *K. pneumoniae* isolates from bloodstream infections between 2012 and 2015 in the region.

## Materials and methods

### Patients and bacterial isolates

This retrospective study was conducted in Ruijin Hospital, an 1800-bed general university-affiliated hospital located in Shanghai, with ~1,15,000 patient visits per year. Patients with at least one positive blood culture of *K. pneumoniae* from January 2012 through December 2015 were enrolled in the study. A total of 254 episodes of *K. pneumoniae* bloodstream infections (66 in 2012, 62 in 2013, 64 in 2014, and 62 in 2015) were identified during this period. Only the first positive blood culture was reviewed and recorded. Eighty isolates were enrolled: twenty isolates were selected from each year using the random number generation function in Microsoft Office Excel 2010 (Microsoft Corporation, Redmond, WA, USA). Isolates identification was carried out using matrix-assisted laser desorption ionization-time of flight mass spectrometer (bioMérieux, Marcy-l'Étoile, France).

This study was approved by Ruijin Hospital Ethics Committee (Shanghai Jiao Tong University School of Medicine). The Review Board exempted request for informed consent because this retrospective study only focused on the bacteria and did not have impact on the patients.

### Antimicrobial susceptibility tests

The antimicrobial susceptibility tests were determined by the disk diffusion method according to the Clinical and Laboratory Standards Institute [Clinical and Laboratory Standards Institute (CLSI), 2015]. The antibiotics tested were ceftazidime, ceftriaxone, cefepime, cefotaxime, cefuroxime, amikacin, gentamicin, tobramycin, piperacillin, aztreonam, ciprofloxacin, levofloxacin, ampicillin-sulbactam, piperacillin-tazobactam, imipenem, meropenem, trimethoprim-sulfamethoxazole, and cefoperazone-sulbactam. *Pseudomonas aeruginosa* ATCC 27853, *K. pneumoniae* ATCC 700603, and *E. coli* ATCC 25922 were used for quality control.

### ESBL-producing and carbapenemase-producing isolates screening and confirmation

According to the CLSI criteria [Clinical and Laboratory Standards Institute (CLSI), 2015], ceftazidime and cefotaxime were used as screening tests for ESBLs. Ceftazidime, ceftazidime-clavulanate, cefotaxime, and cefotaxime-clavulanate were used as confirmatory test. Imipenem and meropenem were used as screening for carbapenemase production. Detection of carbapenemase genes was performed to confirm production of carbapenemases.

### Detection of resistant genes

Polymerase chain reaction (PCR) was performed to amplify the resistant genes using previous primers (Du et al., 2014; Zhao et al., 2016), including ESBL genes (*bla*_TEM_, *bla*_SHV_, *bla*_CTX−M−1, −2, −8, −9, −10, −25 group)_, *bla*_VEB_, *bla*_GES_, *bla*_OXA(−1, −2, −10 group)_, and *bla*_PER_) and carbapenemase genes (*bla*_VIM_, *bla*_IPM_, *bla*_KPC_, *bla*_GIM_, *bla*_SPM_, *bla*_SIM_, *bla*_OXA−48 group_, and *bla*_NDM_). Sample DNA was prepared by boiling the bacteria at 100°C for 15 min, and centrifugation at 3,000 g for 15 min. The PCR conditions used were initial denaturation at 95°C for 5 min, cyclic denaturation at 95°C for 50 s, annealing at 55°C for 30 s, elongation at 72°C for 1 min for 35 cylces and final extension at 72°C for 5 min. PCR products were examined by electrophoresis in 1.5% agarose gel. Positive amplicons were sequenced using ABI3730xl DNAAnalyzer by Sangon Biotech (Shanghai, China) and aligned with subtypes of β-lactamase genes by BLAST (http://blast.ncbi.nlm.nih.gov).

### Multilocus sequence typing

Multilocus sequence typing (MLST) was carried out as described previously (http://bigsdb.web.pasteur.fr/klebsiella/primers_used.html; Diancourt et al., 2005). Briefly, Seven hosekeeping genes (*gapA, infB, mdh, pgi, phoE, rpoB*, and *tonB*) for *K. pneumoniae* were amplified, sequenced, and analyzed. Alleles and STs were determined according to the database (http://bigsdb.web.pasteur.fr/perl/bigsdb/bigsdb.pl?db=pubmlst_klebsiella_seqdef_public&page=profiles). STs that could not be found in the database were submitted to the curator of the database (klebsiellaMLST@pasteur.fr). eBURST version 3.0 software was used to analyze the clustering of related STs. In this study, isolates were grouped together if six of the seven alleles were homologs.

### Statistical analysis

Data in this study were analyzed by SAS 8.2 (SAS Institute Inc., Cary, NC, USA). Continuous variables were presented as the mean ± *SD* or median and interquartile range. For categorical variables, the chi-square test was used to compare the disparity between different groups. *P* < 0.05 was considered to be statistically significant.

## Results

### Patient data

From January 2012 to December 2015, there were more male patients (182/254) than females (72/254). The age of patients ranged from 1 to 91 years. Most of the cases (40/254) were derived from the surgery. Fifty-eight males and 22 females were enrolled and their median age was 61 years (range: 12–91 years). Most patients were from the Department of General Surgery (17/80), Transplantation (11/80), and Infectious Disease (9/80).

### Antimicrobial susceptibility tests

Isolates bear high resistance to cefuroxime (56.3%), ampicillin-sulbactam (52.5%), and piperacillin (52.5%), while there were the least resistance to imipenem (17.5%), meropenem (18.8%), and amikacin (20.0%). Of the 80 isolates, 22 isolates (27.5%) were ESBL producers, and 14 (17.5%) were carbapenemase producers. The resistant rates to most of the antibiotics in ESBL producers were >60%, including cefuroxime (100.0%), piperacillin (100.0%), cefotaxime (100.0%), ceftriaxone (100.0%), ampicillin-sulbactam (86.4%), trimethoprim-sulfamethoxazole (81.8%), gentamicin (77.3%), aztreonam (72.7%), ciprofloxacin (63.6%), and cefepime (63.6%). The resistant rates to antibiotics were significantly higher in ESBL producers than those in non-producers except meropenem, levofloxacin, piperacillin-tazobactam, imipenem, and amikacin. The carbapenemase producers were resistant to all tested drugs except trimethoprim-sulfamethoxazole, tobramycin, amikacin, and gentamicin. Resistant rates to all antibiotics other than trimethoprim-sulfamethoxazole were statistically different between the carbapenemase producers and non-carbapenemase producers (Table 1).

**Table 1:** **Rates of antibiotics resistance in eighty *K. pneumoniae* bloodstream isolates**.

**Antibiotics**	**Number of isolates (%)**			***P***	**Number of isolates (%)**			***P***
	**Total (*n* = 80)**	**ESBL (*n* = 22)**	**Non-ESBL (*n* = 58)**		**Total (*n* = 80)**	**Carbapenemase (*n* = 12)**	**Non-carbapenemase (*N* = 68)**	
Ceftazidime	25 (31.3)	11 (50.0)	14 (24.1)	0.0259	25 (31.3)	12 (100.0)	13 (19.1)	<0.0001
Ceftriaxone	38 (47.5)	22 (100.0)	16 (27.6)	<0.0001	38 (47.5)	12 (100.0)	26 (38.2)	<0.0001
Cefepime	29 (36.3)	14 (63.6)	15 (25.9)	0.0017	29 (36.3)	12 (100.0)	17 (25.0)	<0.0001
Cefotaxime	38 (47.5)	22 (100.0)	16 (27.6)	<0.0001	38 (47.5)	12 (100.0)	26 (38.2)	<0.0001
Cefuroxime	45 (56.3)	22 (100.0)	23 (39.7)	<0.0001	45 (56.3)	12 (100.0)	33 (48.5)	0.0009
Amikacin	16 (20.0)	4 (18.2)	12 (20.7)	1.0000	16 (20.0)	10 (83.3)	6 (8.8)	<0.0001
Gentamicin	33 (41.3)	17 (77.3)	16 (27.6)	<0.0001	33 (41.3)	11 (91.7)	22 (32.4)	0.0004
Tobramycin	26 (32.5)	12 (54.5)	14 (24.1)	0.0095	26 (32.5)	11 (91.7)	15 (22.1)	<0.0001
Piperacillin	42 (52.5)	22 (100.0)	20 (34.5)	<0.0001	42 (52.5)	12 (100.0)	30 (44.1)	0.0004
Aztreonam	31 (38.8)	16 (72.7)	15 (25.9)	0.0001	31 (38.8)	12 (100.0)	19 (27.9)	<0.0001
Ciprofloxacin	33 (41.3)	14 (63.6)	19 (32.8)	0.0122	33 (41.3)	12 (100.0)	21 (30.9)	<0.0001
Levofloxacin	26 (32.5)	9 (40.9)	17 (29.3)	0.3227	26 (32.5)	12 (100.0)	14 (20.6)	<0.0001
Ampicillin-sulbactam	42 (52.5)	19 (86.4)	23 (39.7)	0.0002	42 (52.5)	12 (100.0)	30 (44.1)	0.0004
Piperacillin-tazobactam	20 (25.0)	6 (27.3)	14 (24.1)	0.7725	20 (25.0)	12 (100.0)	8 (11.8)	<0.0001
Imipenem	14 (17.5)	3 (13.6)	11 (19.0)	0.8176	14 (17.5)	12 (100.0)	2 (2.9)	<0.0001
Meropenem	15 (18.8)	2 (9.1)	13 (22.4)	0.2972	15 (18.8)	12 (100.0)	3 (4.4)	<0.0001
Trimethoprim-sulfamethoxazole	38 (47.5)	18 (81.8)	20 (34.5)	0.0002	38 (47.5)	7 (58.3)	31 (45.6)	0.4150

### Characterization of resistance genes

In this study, 40.9% (9/22) *bla*_TEM_, 100.0% (22/22) *bla*_SHV_, and 77.3% (17/22) *bla*_CTX−M_ were identified in the 22 ESBL producers. The dominan ESBL gene was *bla*_CTX−M_. The subtypes of *bla*_TEM_ were *bla*_TEM−1_(*n* = 8) and *bla*_TEM−103_(*n* = 1)_._ The main subtype of *bla*_SHV_ was *bla*_SHV−1_ (*n* = 11), and the most common subtype of *bla*_CTX−M_ was *bla*_CTX−M−14_ (*n* = 7)_._ Other subtypes including *bla*_SHV−33_, *bla*_SHV−36_, *bla*_SHV−11_, *bla*_SHV−12_, *bla*_SHV−28_, *bla*_CTX−M−15_, and *bla*_CTX−M−65_ were also found. Five of the 22 isolates (22.7%) only harbored *bla*_SHV._ While the other 17 isolates additionally harbored one or two ESBL genes, including *bla*_SHV_ along with *bla*_CTX−M_(8/22), and *bla*_SHV_ along with *bla*_CTX−M_ and *bla*_TEM_ (9/22) (Table 2). No *bla*_CTX−M(−2, −8, −10, −25 group)_, *bla*_VEB_, *bla*_GES_, *bla*_OXA(−1, −2, −10 group)_, or *bla*_PER_genes were found.

**Table 2:** **Resistant genes in ESBL-producing or carbapenemase-producing isolates from *K. pneumoniae* bloodstream isolates during 2012–2015**.

**Genes**	**Number of isolates (%)**				
	**Total**	**2012**	**2013**	**2014**	**2015**
ESBL	22 (27.5)	3 (15.0)	7 (35.0)	7 (35.0)	5 (25.0)
*bla*_TEM−1_	8 (36.4)	1 (33.3)	2 (28.6)	2 (28.6)	3 (60.0)
*bla*_TEM−103_	1 (4.5)	1 (33.3)	0 (0.0)	0 (0.0)	0 (0.0)
*bla*_SHV−1_	11 (50.0)	2 (66.7)	5 (71.4)	1 (14.3)	3 (60.0)
*bla*_SHV−33_	1 (4.5)	1 (33.3)	0 (0.0)	0 (0.0)	0 (0.0)
*bla*_SHV−36_	2 (9.1)	0 (0.0)	2 (28.6)	0 (0.0)	0 (0.0)
*bla*_SHV−11_	3 (13.6)	0 (0.0)	0 (0.0)	2 (28.6)	1 (20.0)
*bla*_SHV−12_	4 (18.2)	0 (0.0)	0 (0.0)	4 (57.1)	0 (0.0)
*bla*_SHV−28_	1 (4.5)	0 (0.0)	0 (0.0)	0 (0.0)	1 (20.0)
*bla*_CTX−M−15_	6 (27.3)	1 (33.3)	2 (28.6)	2 (28.6)	1 (20.0)
*bla*_CTX−M−14_	7 (31.8)	0 (0.0)	4 (57.1)	1 (14.3)	2 (40.0)
*bla*_CTX−M−65_	4 (18.2)	2 (66.7)	0 (0.0)	1 (14.3)	1 (20.0)
Carbapenemase	12 (15.0)	0 (0.0)	1 (5.0)	5 (25.0)	6 (30.0)
*bla*_KPC−2_	12 (100.0)	0 (0.0)	1 (100.0)	5 (100.0)	6 (100.0)

The *bla*_KPC−2_ gene was detected in 92.9% (13/14) carbapenemase producers and *bla*_NDM−5_was found in another isolate. No other carbapenemase genes were detected.

### ST and clonal complexes

**Figure 1:**
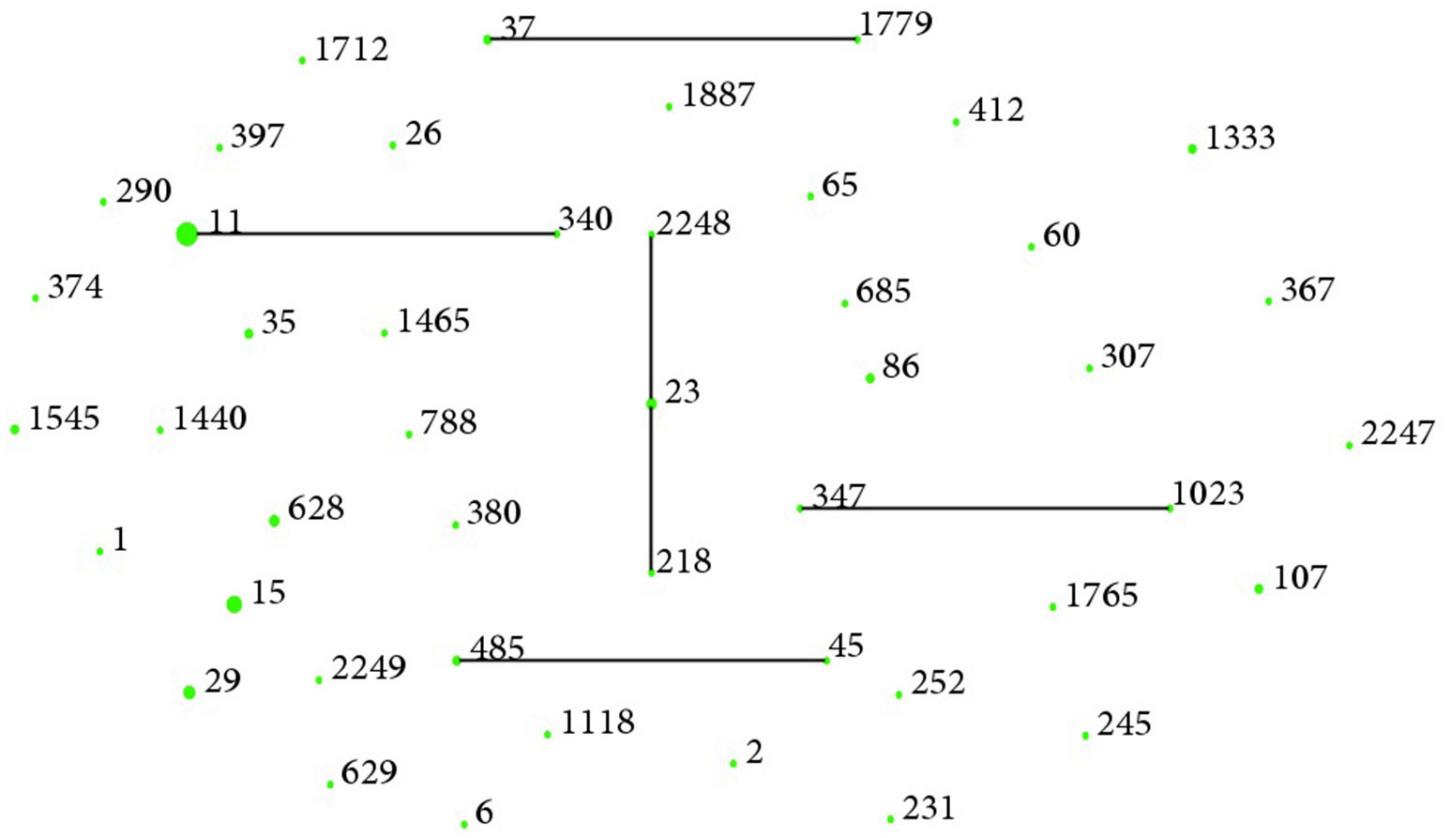
**Clonal groupings among *K. pneumoniae* bloodstream isolates**. There were 35 singletons, four groups (group 1: ST37, ST1779; group 2: ST11, ST340; group 3: ST347, ST1023; group 4: ST485, ST45) and one clonal complexes (ST218, ST23, ST2248) with ST23 as the putative founder. One blob represented one ST and the size reflected the number of isolates belonging to it.

Forty-seven STs among 80 *K. pneumoniae* isolates, including three new STs (ST2247, ST2248, and ST2249) were identified. The most prevalent ST in *K. pneumoniae* isolates was ST11 (*n* = 14, 17.5%), followed by ST15 (*n* = 7, 8.7%), and ST29 (*n* = 4, 5.0%) (Table 3). eBURST indicated that these 47 STs could be clustered into one clonal complex, four groups, and 35 singletons (Figure 1). Notably, ESBL producers were mostly ST15 (5/22, 22.7%), ST11 (3/22, 13.6%), and ST628 (3/22, 13.6%), while all carbapenemase producers belonged to ST11 except the *bla*_NDM−5_-positive one (ST1).

**Table 3:** **Antibiotic resistance profiles and genotypes in MLST of eighty *K. pneumoniae* isolates from bloodstream infections**.

**ST**	**Antibiotic resistance profiles**	**Resistance determinants**	**Number of isolates**
ST1	CAZ-CRO-FEP-CTX-CXM-TOB-PRL-ATM-CIP-LEV-SAM-TZP-IPM-MEM-SXT	–	1
ST2	-	–	1
ST6	CAZ-CRO-FEP-CTX-CXM-PRL-ATM-SAM-TZP	SHV-1	1
ST11	CAZ-CRO-CTX-CXM-TOB-PRL-ATM-CIP-LEV-SAM-SXT	SHV-11	1
	CAZ-CRO-FEP-CTX-CXM-AK-CN-TOB-PRL-ATM-CIP-LEV-SAM-TZP-IPM-MEM	KPC-2	3
	CAZ-CRO-FEP-CTX-CXM-AK-CN-TOB-PRL-ATM-CIP-LEV-SAM-TZP-IPM-MEM	TEM-1, SHV-11, CTX-M-14, KPC-2	1
	CAZ-CRO-FEP-CTX-CXM-AK-CN-TOB-PRL-ATM-CIP-LEV-SAM-TZP-IPM-MEM-SXT	KPC-2	6
	CAZ-CRO-FEP-CTX-CXM-AK-CN-TOB-PRL-ATM-CIP-LEV-SAM-TZP-MEM	–	1
	CAZ-CRO-FEP-CTX-CXM-CN-PRL-ATM-CIP-LEV-SAM-TZP-IPM-MEM-SXT	KPC-2	1
	CAZ-CRO-FEP-CTX-CXM-CN-PRL-ATM-CIP-LEV-SAM-TZP-IPM-MEM-SXT	SHV-12, CTX-M-14, KPC-2	1
	CAZ-CRO-FEP-CTX-CXM-TOB-PRL-ATM-CIP-LEV-SAM-TZP-IPM-MEM		
ST15	CAZ-CRO-FEP-CTX-CXM-AK-CN-TOB-PRL-ATM-CIP-LEV-SAM-IPM-SXT	SHV-1, CTX-M-14	1
	CAZ-CRO-FEP-CTX-CXM-CN-TOB-PRL-ATM-CIP-LEV-SAM-TZP-SXT	SHV-12	1
	CAZ-CRO-FEP-CTX-CXM-CN-TOB-PRL-ATM-CIP-SAM-SXT	TEM-1, SHV-1, CTX-M-15	1
	CAZ-CRO-FEP-CTX-CXM-CN-TOB-PRL-ATM-CIP-SAM-TZP-SXT	TEM-1, SHV-12, CTX-M-15	1
	CIP-LEV-SAM-SXT	–	2
	CRO-CTX-CXM-PRL-ATM-CIP-LEV-SAM-SXT	SHV-1	1
ST23	–	–	3
ST26	CRO-CTX-CXM-PRL-SAM-SXT	SHV-28	1
ST29	–	–	2
	CXM-SAM	–	2
ST35	CTX-CXM-CN-PRL-SXT	–	1
	CAZ-CRO-FEP-CTX-CXM-CN-TOB-PRL-ATM-CIP-SAM-SXT	TEM-1, SHV-33, CTX-M-15	1
ST37	CRO-CXM-CN-PRL-SXT	–	1
	CAZ-CXM-TOB-PRL-CIP-LEV-SAM-SXT	–	1
ST45	–	–	1
ST60	–	–	1
ST65	–	–	1
ST86	CXM-SAM	–	1
	CXM-SXT	–	1
ST107	CRO-FEP-CTX-CXM-CN-PRL-ATM-SAM-SXT	SHV-36, CTX-M-14	2
ST218	–	–	1
ST231	CRO-FEP-CTX-CXM-CN-PRL-ATM-CIP-LEV-SAM-SXT	TEM-1, SHV-1, CTX-M-65	1
ST245	–	–	1
ST252	–	–	1
ST290	CRO-CTX-CXM-CN-PRL-SAM-SXT	TEM-103, SHV-1, CTX-M-65	1
ST307	CAZ-CRO-FEP-CTX-CXM-AK-CN-TOB-PRL-ATM-CIP-LEV-SAM-TZP-SXT	TEM-1, SHV-1, CTX-M-15	1
ST340	CRO-FEP-CTX-CXM-PRL-ATM-CIP-LEV-SAM-TZP-MEM-SXT	–	1
ST347	–	–	1
ST367	–	–	1
ST374	–	–	1
ST380	–	–	1
ST397	CRO-FEP-CTX-CXM-CN-TOB-PRL-ATM-CIP-SAM-SXT	TEM-1, SHV-1, CTX-M-15	1
ST412	–	–	1
ST485	–	–	2
ST628	CRO-CTX-CXM-CN-PRL-SXT	SHV-1, CTX-M-14	1
	CRO-CTX-CXM-CN-PRL-CIP-LEV-SAM-SXT	SHV-1, CTX-M-14	1
	CRO-CTX-CXM-CN-PRL-SXT	SHV-1, CTX-M-65	1
ST629	CRO-CTX-CXM-CN-TOB-PRL	SHV-11, CTX-M-65	1
ST685	CIP-LEV-SXT	–	1
ST788	–	–	1
ST948	–	–	1
ST1023	CXM-AK-CN-TOB-PRL-CIP-SAM-SXT	–	1
ST1118	SXT	–	1
ST1333	–	–	2
ST1440	–	–	1
ST1465	CAZ-CRO-FEP-CTX-CXM-AK-CN-TOB-PRL-ATM-CIP-SAM-SXT	TEM-1, SHV-12, CTX-M-15	1
ST1545	–	–	2
ST1712	CRO-FEP-CTX-CXM-CN-PRL-ATM-CIP-SAM-SXT	–	1
ST1765	CAZ-CRO-FEP-CTX-CXM-AK-CN-TOB-PRL-ATM-SAM-TZP	–	1
ST1779	PRL-SAM	–	1
ST1887	SXT	–	1
ST2247	–	–	1
ST2248	–	–	1
ST2249	–	–	1

*CAZ, ceftazidime; CRO, ceftriaxone; FEP, cefepime; CTX, cefotaxime; CXM, cefuroxime; AK, amikacin; CN, gentamicin; TOB, tobramycin; PRL, piperacillin; ATM, aztreonam; CIP, ciprofloxacin; LEV, levofloxacin; SAM, ampicillin-sulbactam; TZP, piperacillin-tazobactam; IPM, imipenem; MEM, meropenem; SCF, cefoperazone-sulbactam; SXT, trimethoprim-sulfamethoxazole*.

## Discussion

Local epidemiologic data on prevalence of specific clones of *K. pneumoniae* bloodstream infection were indispensible to develop clinical treatment regimen and evaluate outcomes of different therapeutic strategy (Pai et al., 2004; Marra et al., 2006; Neuner et al., 2011; Harris et al., 2015). However, there were limited data on antibiotic resistance, resistant genes and STs of *K. pneumoniae* bloodstream isolates in Shanghai. In our pilot study conducted in 2012, we found that *K. pneumoniae* was the second frequent gram negative bacillus from blood cultures in our hospital, representing 14.3% of all the isolates (Zhao et al., 2014). In current study, we have extended our research through 2015 to acquire more comprehensive molecular epidemiologic data.

*K. pneumoniae* bloodstream isolates in the study showed threatened resistance to the most of routine antibiotics and only meropenem, imipenem, amikacin, and piperacillin-tazobactam had relative low resistant rates (<30%) which made them better candidates for empiric therapy of *K. pneumoniae* bloodstream infections. However, prudent and rational uses of antibiotics based on the results of antimicrobial susceptibility tests are still essential to the success of treatment. Notably, the resistant rates to meropenem, imipenem, amikacin, cefepime, and ciprofloxacin determined in this study were much higher than what were found in the surveillance of 2011–2012 in China (Li et al., 2014). This dynamic change of the pattern of drug resistance warrant active ongoing surveillance on antibiotic resistance and consistent prevention and control of *K. pneumoniae*.

The proportion of ESBL-producing *K. pneumoniae* (27.5%) in this study was lower than that in Italy (32.6%), America (51.8%), Korea (52.9%), and Russia (60.8%) (Kim et al., 2002; Edelstein et al., 2003; Marra et al., 2006; Tumbarello et al., 2006), but the multidrug-resistant phenotype, and the dominate ESBL enzyme (CTX-M) of ESBL producers were similar (Edelstein et al., 2003). Typing of ESBL producers revealed a high level of genetic diversity, with ST15 (22.7%, 5/22) and ST11 (13.6%, 3/22) as the most common STs. ST15 *K. pneumoniae* has been identified in both animals and humans in several countries. Although it was not yet a dominant clone in our study, ST15 *K. pneumoniae* had achieved a highly successful clonal spread in some countries, such as Bulgaria, Portugal, and Thailand (Netikul et al., 2014; Rodrigues et al., 2014; Markovska et al., 2015), and we should be cautious about its high potential of becoming a major clone associated with ESBL producers in Shanghai in future. Isolates belonged to ST11 were isolated from different departments, in different years with different antibiotic resistance profiles. This suggested no clonal dissemination in the region.

Different from previous studies where no carbapenemase-producing *E. coli* bloodstream isolate was found (Zhao et al., 2015; Wang et al., 2016), the carbapenemase-producing *K. pneumoniae* accounted for 17.5% in our hospital, and they, as reported elsewhere (David et al., 2013; Du et al., 2014), also showed extremely high resistant rates to major antibiotics except aminoglycosides and trimethoprim-sulfamethoxazole. Molecular analysis suggested 92.9% of the carbapenemase producers were harbored with *bla*_KPC−2_, and belonged to ST11, confirming that ST11 was associated with KPC. Unlike the widespread of KPC-producing ST258 *K. pneumoniae* in Europe, the dominant ST of KPC-producing *K. pneumoniae* was ST11 in China (Dautzenberg et al., 2016). Also, One ST1 carbapenemase producers carried *bla*_NDM−5_ was found in this study. NDM-5, which mostly found in *E. coli*, was firstly reported in *K. pneumoniae* in Shanghai. Taken together, our data indicated the KPC-producing ST11 *K. pneumoniae* isolate was a high-risk clone in our hospital, and should be taken as the major consideration when developing strategy to control resistant isolates dissemination. NDM, along with KPC, VIM, and OXA-48, were four major carbapenemases detected in *K. pneumoniae*. The most common type of NDM found was NDM-1. NDM-5, an emerging carbapenemase in *K. pneumoniae*, should attract our attention.

Our study described the phenotypic and molecular properties of *K. pneumoniae* bloodstream isolates in Shanghai for the first time. This study also suggested ST11 *K. pneumoniae* harbored *bla*_KPC−2_ had absolute predominance in carbapenemase producers, and NDM-5 was an emerging carbapenemase. Although our conclusion based on a single hospital cannot be directly extrapolated to the whole area, it provides the stepstone for the future expanded research associated with multicenter and further resistant mechanism surveillance to prevent further possible dissemination in this region.

## Author contributions

Conceived and designed the experiments: LH, SX, SW, and SZ. Performed the experiments: SW and SX. Analyzed the data: SX and SW. Contributed reagents/materials/analysis tools: LH, YN, and XG. Wrote the paper: SX, SW, LH, WW, FG, and JQ.

## Funding

This work was supported by Special Fund for Health-scientific Research in the Public Interest: Research & application for the prevention & control of nosocomial infections caused by multi-drug resistant bacteria (201002021) and The Shanghai 3-Year Plan of the Key Subjects Construction in Public Health-Infectious Diseases and Pathogenic Microorganism (15GWZK0102). The funders had no role in study design, data collection and analysis, decision to publish, or preparation of the manuscript.

### Conflict of interest statement

The authors declare that the research was conducted in the absence of any commercial or financial relationships that could be construed as a potential conflict of interest.

## References

[B1] Biedenbach D. J. Moet G. J. Jones R. N. (2004). Occurrence and antimicrobial resistance pattern comparisons among bloodstream infection isolates from the SENTRY Antimicrobial Surveillance Program (1997–2002). Diagn. Microbiol. Infect. Dis. 50, 59–69. 10.1016/j.diagmicrobio.2004.05.003 15380279

[B2] Clinical Laboratory Standards Institute (CLSI) (2015). Performance Standards for Antimicrobial Susceptibility Testing[S]: Twenty-fourth Informational Supplement. Wayne, PA: Clinical and Laboratory Standards Institute.

[B3] Daikos G. L. Tsaousi S. Tzouvelekis L. S. Anyfantis I. Psichogiou M. Argyropoulou A.. (2014). Carbapenemase-producing *Klebsiella pneumoniae* bloodstream infections: lowering mortality by antibiotic combination schemes and the role of Carbapenems. Antimicrob. Agents Chemother. 58, 2322–2328. 10.1128/AAC.02166-13 24514083 PMC4023796

[B4] Dautzenberg M. J. Haverkate M. R. Bonten M. J. Bootsma M. C. (2016). Epidemic potential of *Escherichia coli* ST131 and *Klebsiella pneumoniae* ST258: a systematic review and meta-analysis. BMJ Open 6:e009971. 10.1136/bmjopen-2015-009971 26988349 PMC4800154

[B5] David V. D. Keith S. K. Elizabeth A. N. Robert A. B. (2013). Carbapenem-resistant Enterobacteriaceae: a review of treatment and outcomes. Diagn. Microbiol. Infect. Dis. 75, 6 10.1016/j.diagmicrobio.2012.11.009 PMC3947910 23290507

[B6] Diancourt L. Passet V. Verhoef J. Grimont P. A. Brisse S. (2005). Multilocus sequence typing of *Klebsiella pneumoniae* nosocomial isolates. J. Clin. Microbiol. 43, 4178–4182. 10.1128/JCM.43.8.4178-4182.2005 16081970 PMC1233940

[B7] Du J. Li P. Liu H. Lü D. Liang H. Dou Y. (2014). Phenotypic and molecular characterization of multidrug resistant *Klebsiella pneumoniae* isolated from a University Teaching Hospital, China. PLoS ONE 9:e95181. 10.1371/journal.pone.0095181 24740167 PMC3989316

[B8] Edelstein M. Pimkin M. Palagin I. Edelstein I. Stratchounski L. (2003). Prevalence and molecular epidemiology of CTX-M extended-spectrum -lactamase-producing *Escherichia coli* and *Klebsiella pneumoniae* in Russian Hospitals. Antimicrob. Agents Chemother. 47, 3724–3732. 10.1128/AAC.47.12.3724-3732.2003 14638473 PMC296190

[B9] Ghadiri H. Vaez H. Khosravi S. Soleymani E. (2012). The antibiotic resistance profiles of bacterial strains isolated from patients with hospital-acquired bloodstream and urinary tract infections. Crit. Care Res. Pract. 2012:890797. 10.1155/2012/890797 23304471 PMC3530749

[B10] Guana J. Zhuo C. DH S. (2014). CHINET 2012 surveillance of antibiotic resistance in *Klebsiella* spp in China. Chin. J. Infect. Chemother. 14, 398–404. 10.3969/j.issn.1009-7708.2014.05.006

[B11] Harris P. N. A. Yin M. Jureen R. Chew J. Ali J. Paynter S. (2015). Comparable outcomes for β-lactam/β-lactamase inhibitor combinations and carbapenems in definitive treatment of bloodstream infections caused by cefotaxime-resistant *Escherichia coli* or *Klebsiella pneumoniae*. Antimicrob. Resist. Infect. Control 4:14 10.1186/s13756-015-0055-6 25932324 PMC4414382

[B12] Hu F. Zhu D. Wang F. (2015). CHINET 2014 surveillance of bacterial resistance in China. Chin. J. Infect. Chemother. 15, 401–410. 10.3969/j.issn.1009-7708.2015.05.001

[B13] Kim Y. K. Pai H. Lee H. J. Park S. E. Choi E. H. Kim J.. (2002). Bloodstream infections by extended-spectrum β-lactamase-producing *Escherichia coli* and *Klebsiella pneumoniae* in children: epidemiology and clinical outcome. Antimicrob. Agents Chemother. 46, 1481–1491. 10.1128/AAC.46.5.1481-1491.2002 11959586 PMC127143

[B14] Li Y. Lv Y. Xue F. (2014). Antimicrobial susceptibility surveillance of gram–negative bacterial from Mohnarin 2011–2012. Chin. J. Clin. Pharmacol. 30, 260–277.

[B15] Lv Y. Li Y. Xue F. (2014). Mohnarin report of 2011–2012: surveillance for resistance of bacteria causing bloodstream infections. Chin. J. Clin. Pharmacol. 30, 278–288.

[B16] Markovska R. Stoeva T. Schneider I. Boyanova L. Popova V. Dacheva D.. (2015). Clonal dissemination of multilocus sequence type ST15 KPC-2-producing *Klebsiella pneumoniae* in Bulgaria. APMIS 123, 887–894. 10.1111/apm.12433 26303718

[B17] Marra A. R. Wey S. B. Castelo A. Gales A. C. Cal R. G. R. Filho J. C.. (2006). Nosocomial bloodstream infections caused by *Klebsiella pneumoniae*: impact of extended-spectrum β-lactamase (ESBL) production on clinical outcome in a hospital with high ESBL prevalence. BMC Infect. Dis. 6:24. 10.1186/1471-2334-6-24 16478537 PMC1382232

[B18] Netikul T. Sidjabat H. E. Paterson D. L. Kamolvit W. Tantisiriwat W. Steen J. A. (2014). Characterization of an IncN2-type blaNDM-(1)-carrying plasmid in *Escherichia coli* ST131 and *Klebsiella pneumoniae* ST11 and ST15 isolates in Thailand. J. Antimicrob. Chemother. 69, 3161–3163. 10.1093/jac/dku275 25096073

[B19] Neuner E. A. Yeh J. Y. Hall G. S. Sekeres J. Endimiani A. Bonomo R. A.. (2011). Treatment and outcomes in Carbapenem-resistant *Klebsiella pneumoniae* bloodstream infections. Diagn. Microbiol. Infect. Dis. 69, 357–362. 10.1016/j.diagmicrobio.2010.10.013 21396529 PMC3058153

[B20] Pai H. Kang C. I. Byeon J. H. Lee K. D. Park W. B. Kim H. B.. (2004). Epidemiology and clinical features of bloodstream infections caused by AmpC-Type-β-lactamase-producing *Klebsiella pneumoniae*. Antimicrob. Agents Chemother. 48, 3720–3728. 10.1128/AAC.48.10.3720-3728.2004 15388426 PMC521917

[B21] Rodrigues C. Machado E. Ramos H. Peixe L. Novais A. (2014). Expansion of ESBL-producing *Klebsiella pneumoniae* in hospitalized patients: a successful story of international clones (ST15, ST147, ST336) and epidemic plasmids (IncR, IncFIIK). Int. J. Med. Microbiol. 304, 1100–1108. 10.1016/j.ijmm.2014.08.003 25190354

[B22] Tumbarello M. Spanu T. Sanguinetti M. Citton R. Montuori E. Leone F.. (2006). Bloodstream infections caused by Extended-spectrum-β-lactamase-producing *Klebsiella pneumoniae*: risk factors, molecular epidemiology, and clinical outcome. Antimicrob. Agents Chemother. 50, 498–504. 10.1128/AAC.50.2.498-504.2006 16436702 PMC1366869

[B23] Wang S. Zhao S. Y. Xiao S. Z. Gu F. F. Liu Q. Z. Tang J.. (2016). Antimicrobial resistance and molecular epidemiology of *Escherichia coli* causing bloodstream infections in three hospitals in Shanghai, China. PLoS ONE 11:e0147740. 10.1371/journal.pone.0147740 26824702 PMC4733056

[B24] Yang Z. Zhan S. Wang B. (2010). Fatality anf secular trend of bloodstream infections during hospitalization in China: a systematie review and meta-analysis. J. Peking Univ. (Health Sciences) 42, 304–307. 20559406

[B25] Zhao S. Y. Wang Y. C. Xiao S. Z. Jiang X. F. Guo X. K. Ni Y. X.. (2015). Drug susceptibility and molecular epidemiology of *Escherichia coli* in bloodstream infections in Shanghai, China, 2011–2013. Infect. Dis. 47, 310–318. 10.3109/00365548.2014.990509 25712794

[B26] Zhao S. Y. Xiao S. Z. Han L. Z. Mi C. R. Ni Y. X. (2014). Distribution and antimicrobial resistance of pathogens isolated from hospitalized patients with bloodstream infections Chin. J. Infect. Control 13, 266–270. 10.3969/j.issn.1671-9638.2014.05.003

[B27] Zhao S. Y. Zhang J. Zhang Y. L. Wang Y. C. Xiao S. Z. Gu F. F.. (2016). Epidemiology and risk factors for faecal extended-spectrum β-lactamase-producing Enterobacteriaceae (ESBL-E) carriage derived from residents of seven nursing homes in western Shanghai, China. Epidemiol. Infect. 144, 695–702. 10.1017/s0950268815001879 26260355

